# Alternations of White Matter Structural Networks in First Episode Untreated Major Depressive Disorder with Short Duration

**DOI:** 10.3389/fpsyt.2017.00205

**Published:** 2017-10-25

**Authors:** Yi Lu, Zonglin Shen, Yuqi Cheng, Hui Yang, Bo He, Yue Xie, Liang Wen, Zhenguang Zhang, Xuejin Sun, Wei Zhao, Xiufeng Xu, Dan Han

**Affiliations:** ^1^Department of Medical Imaging, The First Affiliated Hospital, Kunming Medical University, Kunming, China; ^2^Department of Psychiatry, The First Affiliated Hospital, Kunming Medical University, Kunming, China; ^3^Biomedical Engineering Research Center, Kunming Medical University, Kunming, China

**Keywords:** major depressive disorder, short duration, structural network, graph theory, network-based statistical

## Abstract

It is crucial to explore the pathogenesis of major depressive disorder (MDD) at the early stage for the better diagnostic and treatment strategies. It was suggested that MDD might be involving in functional or structural alternations at the brain network level. However, at the onset of MDD, whether the whole brain white matter (WM) alterations at network level are already evident still remains unclear. In the present study, diffusion MRI scanning was adopt to depict the unique WM structural network topology across the entire brain at the early stage of MDD. Twenty-one first episode, short duration (<1 year) and drug-naïve depression patients, and 25 healthy control (HC) subjects were recruited. To construct the WM structural network, atlas-based brain regions were used for nodes, and the value of multiplying fiber number by the mean fractional anisotropy along the fiber bundles connected a pair of brain regions were used for edges. The structural network was analyzed by graph theoretic and network-based statistic methods. Pearson partial correlation analysis was also performed to evaluate their correlation with the clinical variables. Compared with HCs, the MDD patients had a significant decrease in the small-worldness (σ). Meanwhile, the MDD patients presented a significantly decreased subnetwork, which mainly involved in the frontal–subcortical and limbic regions. Our results suggested that the abnormal structural network of the orbitofrontal cortex and thalamus, involving the imbalance with the limbic system, might be a key pathology in early stage drug-naive depression. And the structural network analysis might be potential in early detection and diagnosis of MDD.

## Introduction

Major depressive disorder (MDD) is one of the most prevalent psychiatric disorders worldwide, with complex manifestations including affected mood, cognitive deficits, and psychomotor disturbances (1). It has affected more than 350 million people globally (2) and has been ranked as the second leading cause of worldwide disability (3). Approximately 75% of MDD patients experience more than one clinically significant episode in their lifetimes (4, 5). Early studies suggested that recurrent episodes and prolonged duration may severely interfere with individual life quality, reduce the effectiveness of antidepressant medication, and greatly increase the risk of suicide (6, 7). Earlier treatments could help to reduce the recurrence rate of depression (8). However, as a disease with high heterogeneity, the exact pathogenesis of MDD remains unclear, the precise biomarker for early diagnosis is still not available. Therefore, it is important to investigate the brain imaging based biomarkers for potential early diagnosis, to improve the rate of success in treatment.

In recent years, functional and structural MRI analyses have been widely applied to non-invasively investigate the brain regions involved in the pathogenesis of depression (9). Most studies have consistently reported functional or structural alterations in various brain regions in MDD patients mainly involved in the fronto-limbic circuit, such as amygdala, hippocampus, cingulate cortex, and prefrontal cortex (10, 11). The volume reduction in striatum and thalamus was also found in MDD patients (12–14). Depression was considered to be implicated in the abnormality of limbic-cortical-striatial-pallidal-thalamic (LCSPT) network (15, 16) or limbic-cortico-striatal-thalamic-cortical (LCSTC) circuits (17). Besides, convergent evidence indicates the default mode network also plays an important role in the pathogenesis of depression (18–22).

Functional or structural abnormalities of these circuits or networks suggest that depression is a complex disorder that mainly involved in the brain network-level alternations, rather than the impairments of isolated regions. However, most published functional or structural MRI studies just provide a limited window into a whole systems level understanding of the abnormalities present in depression (23). The recent explosive growth of connectome approaches has made it possible to quantify the topological structural organization of complex neural networks across the entire brain (24–26). Graph theoretic and network-based statistic (NBS) analyses were the two general methods used in the whole-brain network analysis. Recent studies have point out the importance of topological disturbances of whole-brain networks as the pathogenesis of MDD (27). Until now, only a few studies investigated the topology of the whole-brain’s white matter (WM) structural network in MDD patients by diffusion MRI. In the graph theoretic analysis, although several structural network studies reported negative group differences in the network measures between the whole MDD and healthy control (HC) groups (28, 29), there were some differences found in the part of the network measures (i.e., clustering coefficient, *C*_p_; characteristic path length, *L*_p_; normalized clustering coefficient, γ; normalized characteristic path length, λ; small-worldness, σ; global efficiency, *E*_global_; local efficiency, *E*_local_) in MDD subgroups, like the early adult-onset and geriatric depression patients (30, 31). Meanwhile, in the NBS analysis, although the above mentioned studies consistently show the structural network alternations among the frontal lobe, striatum and limbic system, there were still some differences in the range of structural network alternations among these studies. These varied findings may be related to certain confounding factors in the MDD patients, such as the number of episodes, age of onset, medication status, and durations (27).

This study aims to explore the unique structural network topology across the entire brain at the early stage of MDD and to elucidate their relationships with clinical severities. Therefore, we only recruited first episode, untreated and short duration MDD patients. We also combined both graph theoretic and network-based statistical analysis together to investigate the structural networks in MDD patients and healthy individuals. Besides, in order to better evaluate the WM structural network, we define the network edges as the multiplication of fiber number (FN) by the mean fractional anisotropy (FA) along the fiber bundles between a pair of cortical regions.

## Materials and Methods

### Subjects

This was a cross-sectional study and all protocols were approved by the ethics committee of Kunming Medical University, and all patients involved in the study provided written informed consent.

In this study, 23 first episode, short duration (<1 year, range = 1–10 months) and drug-naive patients were recruited from the psychiatry department of the First Affiliated Hospital of Kunming Medical University. And two of the 23 patients were excluded due to obvious head motion. Ultimately, 21 patients (8 women and 13 men, age range 18–56 years; 100% right handed; education years range = 12–20) were recruited. Two experienced psychiatrists independently made the diagnosis of MDD according to the diagnostic assessment using the Structured Clinical Interview for DSM-IV-Patient Edition (SCID-P). All of the MDD patients also had a score of 18 or greater (scores range: 18–34) on the 17-item Hamilton Depression Rating Scale (HDRS). Patients that had other comorbid Axis I and Axis II psychiatric disorders, such as schizophrenia, bipolar affective disorder, and personality disorders, were excluded from this study according to the SCID-I and SCID-II assessments. Although some MDD patients had anxiety symptom, they did not meet the diagnostic criteria for anxiety disorders. The MDD patients included in the study had never received antidepressive medications before the MRI examinations.

A total of 25 HC subjects matched for age, gender, and education years were also recruited from Kunming. They were screened using a diagnostic interview, the Structured Clinical Interview for DSM-IV Nonpatient Edition (SCID-NP), to rule out current or past DSM-IV Axis I disorders. They were also interviewed to affirm that there was no history of psychiatric illness in their first-degree relatives. All subjects were right-handed and without severe or acute medical conditions physically based on clinical evaluations and medical records. All of the HC subjects involved in the study provided written informed consent.

### Power Analysis

On the basis of preliminary experiments and previous study, a difference mean of 0.32 with 0.37 SD for the clustering coefficient (Cp) between MDD patients and health control, which was most mentioned by previous studies, was hypothesized; And the sample size of patients in several previous researches was probably around 22 (30–32). So a sample size of 21 patients would have an 80% power to detect such a difference as statistically significant at a level (α) of 0.05 in the present study.

### Magnetic Resonance Imaging Acquisition

All participants were scanned on a Philips 3 T achieva TX scanner with an eight-channel head coil. A diffusion tensor image sequence was applied with the following parameters: TR = 7,173 ms, TE = 78 ms, matrix = 115 × 115, FOV = 230 mm × 230 mm, 50 axial slices, b value = 1,000, directions = 32, slice thickness = 3 mm, acquisition time = 9 min 7 sec. A high-resolution 3D TFE sequence was acquired with the following parameters: TR = 7.7 ms, TE = 3.6 ms, matrix = 228 × 228, FOV = 250 mm × 250 mm, 230 axial slices, acquisition time = 6 min 53 sec. In addition, axial T2-weighted MR images were acquired with the parameters: TR = 2,500 ms, TE = 80 ms, matrix = 332 × 225, FOV = 250 mm × 220 mm, slice thickness = 6 mm, 18 axial slices, acquisition time = 55 sec. The anatomical MR images were re-evaluated for any structural abnormalities and were reported as normal in all subjects.

### Structural Brain Network Construction

In this study, DTI data preprocessing and brain network were performed using the PANDA toolbox (33)^1^ which was an integration analysis toolbox comprising the Diffusion Toolkit (34), FMRIB Software Library (35), MRIcron (36), and Pipeline System for Octave and Matlab (PSOM) (37). And the whole process had been described in detail previously (38).

#### DTI Data Preprocessing

Briefly, the preprocessing procedure included skull-stripping, eddy-current, and head-motion correction, FA calculation and whole-brain deterministic DTI fiber tractography (39). First, the non-brain tissues of the images were deleted by employing the brain extraction tool (40). Second, the head motion and eddy current distortions were corrected through registering the DW images to the b0 image with an affine transformation (41). After correction, six elements of the diffusion tensor were then estimated from which FA was calculated. Then, Whole-brain fiber tractography was subsequently reconstructed by seeding at every voxel in the brain and using fiber assignment by continuous tracking algorithm (42). This algorithm computes fiber trajectories starting from the deep WM regions and terminating at a voxel with a turning angle greater than 45° or reached a voxel with FA less than 0.15.

As the construction of the structural network requires the following basic elements: nodes and edges, we adopted the same procedures used in previous WM network studies to define network nodes and edges.

#### Network Node Definition

In this study, we parcellated the cerebral cortex into 90 cortical and subcortical regions (45 for each hemisphere, see Table S1 in Supplementary Material), using the Automated Anatomical Labeling (AAL) template. And each region representing a node of the cortical network (26). For each participant, the parcelation process must be conducted in the native DTI space. To achieve this, the individual 3D T1-weighted images were coregistered to the b0 images in the DTI native space. The transformed 3D T1-weighted images were then nonlinearly transformed to the ICBM152 T1 template in the Montreal Neurological Institute (MNI) space. Inverse transformations were used to warp the AAL atlas from the MNI space to the DTI native space (43).

#### Network Edge Definition

The brain structural network was constructed by combining the WM tractography with the individual parcelation map for each subject. To define the network edges, we computed the edge weight (*w_ij_*) as the multiplication of FN by the mean FA along the fiber bundles between a pair of cortical regions, *w_ij_* = FN*_ij_* × FA*_ij_* (44). To avoid the influence of spurious connections, all edges with FN of <3 were set to zero. Following the steps above, we constructed a symmetric weighted structural brain network (90 × 90) for each participant.

### Graph Theoretical Analysis of Structural Brain Networks

To better characterize the brain structural network topology in the present study, we adopted graph theoretical analysis to provide quantitative metrics to describe any difference in brain structural network topology between MDD patients and HCs. Meanwhile, the whole-brain structural network topological organization can be systematically studied at both global and regional levels. For the comparison of global network properties across participants and groups, we used a sparsity (connection density) threshold (*S*), which retains *S*% of the top connections for each participant. This threshold ensured that the number of nodes and connections were matched across participants (28). To avoid the influence of biases caused by single threshold, we examined topological properties across a range of thresholds (5% < Sparsity < 40%, in steps of 1%). Global network architecture was quantified in terms of small-world properties (small-worldness, σ; normalized characteristic path length, λ; normalized clustering coefficient, γ; characteristic path length, *L*_p_; clustering coefficient, *C*_p_) and efficiency (local efficiency, *E*_local_; global efficiency, *E*_global_). And the behavior of each node was also described with nodal efficiency (*E*_nodal_). For these network measurements, we computed the area under the curve across the full range of sparsity thresholds for comparison between MDD and HCs groups. A univariate analysis of covariance was then used to assess the group effects. Age was considered as a nuisance covariate and thus regressed out. To correct the false-positive error caused by multiple comparisons, an additional false discovery rate (FDR) correction was applied for these comparisons. The significance level was set at *p* < 0.05. All network properties analysis and statistical analysis were performed using the GRETNA toolbox (45)^2^ and visualized by using the BrainNet Viewer toolbox (46).^3^

### NBS Analysis

To assess differences in the interregional connectivity matrix between the MDD and control groups, we used a recently developed NBS approach (47). The NBS was implemented in the present study as following steps below: First, a two-sample *t*-statistic was calculated for each pair of regions of the AAL template to test the null hypothesis of equality in the mean value of structural connectivity between groups. Second, the connections exceeding the set threshold of 2.1 were considered as suprathreshold connections. And then, topological clusters among the suprathreshold connections were identified. Finally, a family wise error corrected *p* value was ascribed to each network using non-parametric permutation testing (5,000 permutations). Subnetworks with a corrected level of *p* < 0.05 were reported. All procedures mentioned above were performed using the NBS Toolbox (http://www.nitrc.org/projects/nbs). And the significant subnetworks were also visualized by using BrainNet viewer.

### Correlations between Network Measures and Clinical Variables

For the network measurement results, which significantly different between the MDD and control groups (i.e., nodes, edges), a Pearson partial correlation analysis was performed to evaluated their association with the clinical variables (i.e., HDRS scores, illness duration) in the MDD group after controlling for the effects of age (*p* < 0.05). Statistical analyses were conducted using IBM SPSS Statistics (version 17.0; IBM, Armonk, NY, USA).

## Results

### Demographics and Clinical Information

Clinical data of 21 right-handed MDD patients and 25 age- and gender-matched health controls (HCs) are presented in Table 1. The two groups did not differ in age (*p* = 0.091), gender (*p* = 0.665) and number of years of education (*p* = 0.193). The mean HDRS score of the MDD patients was 24.38 ± 4.08 (range = 18–34) and the mean duration was 3.47 ± 2.60 months (range = 1–10 months). Besides, the mean HARS scores was 24.47 ± 8.38. Data are expressed as mean ± SD. An unpaired *t*-test and Pearson chi-square test were performed, respectively, in the comparison of the continuous variables and gender.

**Table 1:** Clinical data of MDD patients and control subjects.

	MDD patients (*n* = 21)	HCs (*n* = 25)	*p*
Age (years)	37.5 ± 11.57	31.4 ± 10.96	0.091 > 0.05
Gender (female/male)	8/13	8/17	0.665 > 0.05
Hand (left/right)	0/21	0/25	
Education years	14.71 ± 3.40	16.16 ± 3.91	0.193 > 0.05
HDRS scores	24.38 ± 4.08		
Duration (months)	3.47 ± 2.60		
HARS scores	24.47 ± 8.38		

*Data are expressed as mean ± SD*.

*Unpaired t-test for the continuous variables and Pearson chi-square test for the gender were performed using the Statistical Package for Social Sciences (SPSS) (version 17.0; IBM, Armonk, NY, USA)*.

*MDD, major depressive disorder; HCs, healthy controls; HDRS, Hamilton Depression Rating Scale; HARS, Hamilton Anxiety Rating Scale*.

### Alterations of Global Network Measures

**Figure 1:**
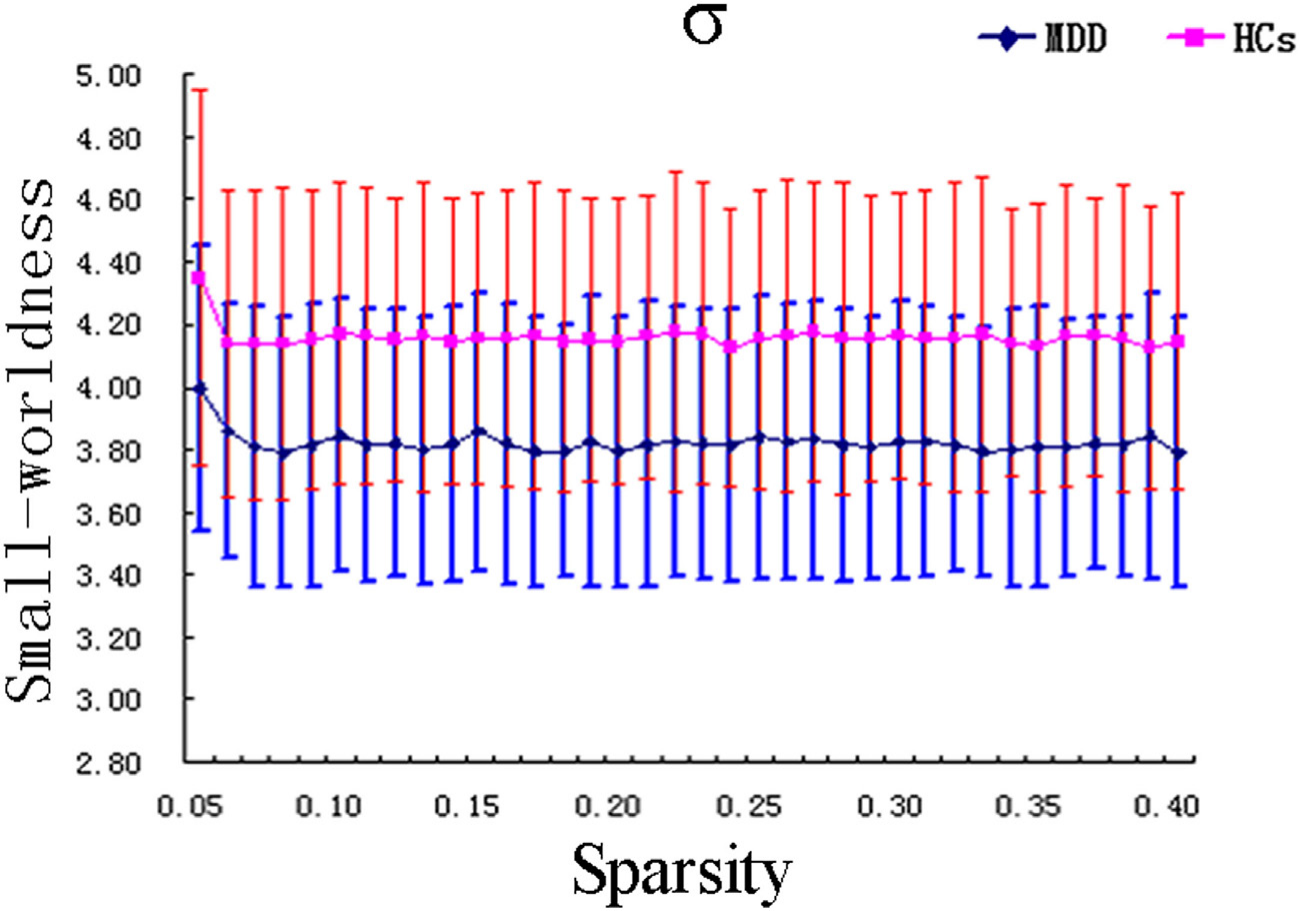
Graph theoretical analysis showed the MDD patients had statistically significant lower small-worldness (FDR corrected, *p* < 0.05). σ, small-worldness; MDD, major depressive disorder; HCs, healthy controls.

Within the scope of the applied network sparsity, we observed that both MDD and HC groups exhibited small-world characteristics (MDD group: σ = 3.82 ± 0.43 > 1; HC group: σ = 4.16 ± 0.48 > 1) (Figure 1). However, when compared with controls, MDD patients showed a significantly differences in the small-worldness parameters (FDR corrected, *p* = 0.029 < 0.05, *t* = −2.253). No significant difference was found in other global network measures including *C*_p_, *L*_p_, γ, λ, *E*_glob_, and *E*_loc_ between the MDD patients and controls (see Figure S1 and Table S2 in Supplementary Material).

### Alterations of Regional Network Measures

In regional network measures, there is no region showing difference survived after FDR correction between the MDD patients and HCs. But, at the uncorrected level, the MDD group showed a nodal efficiency reduction in the left orbital part of middle frontal gyrus (ORBmid.L, *p* = 0.029 < 0.05, uncorrected, *t* = −2.256) and thalamus (THA.L, *p* = 0.026 < 0.05, uncorrected, *t* = −2.295) (see Figure S2 in Supplementary Material).

### Whole-brain Mapping of Connectivity Alterations

**Figure 2:**
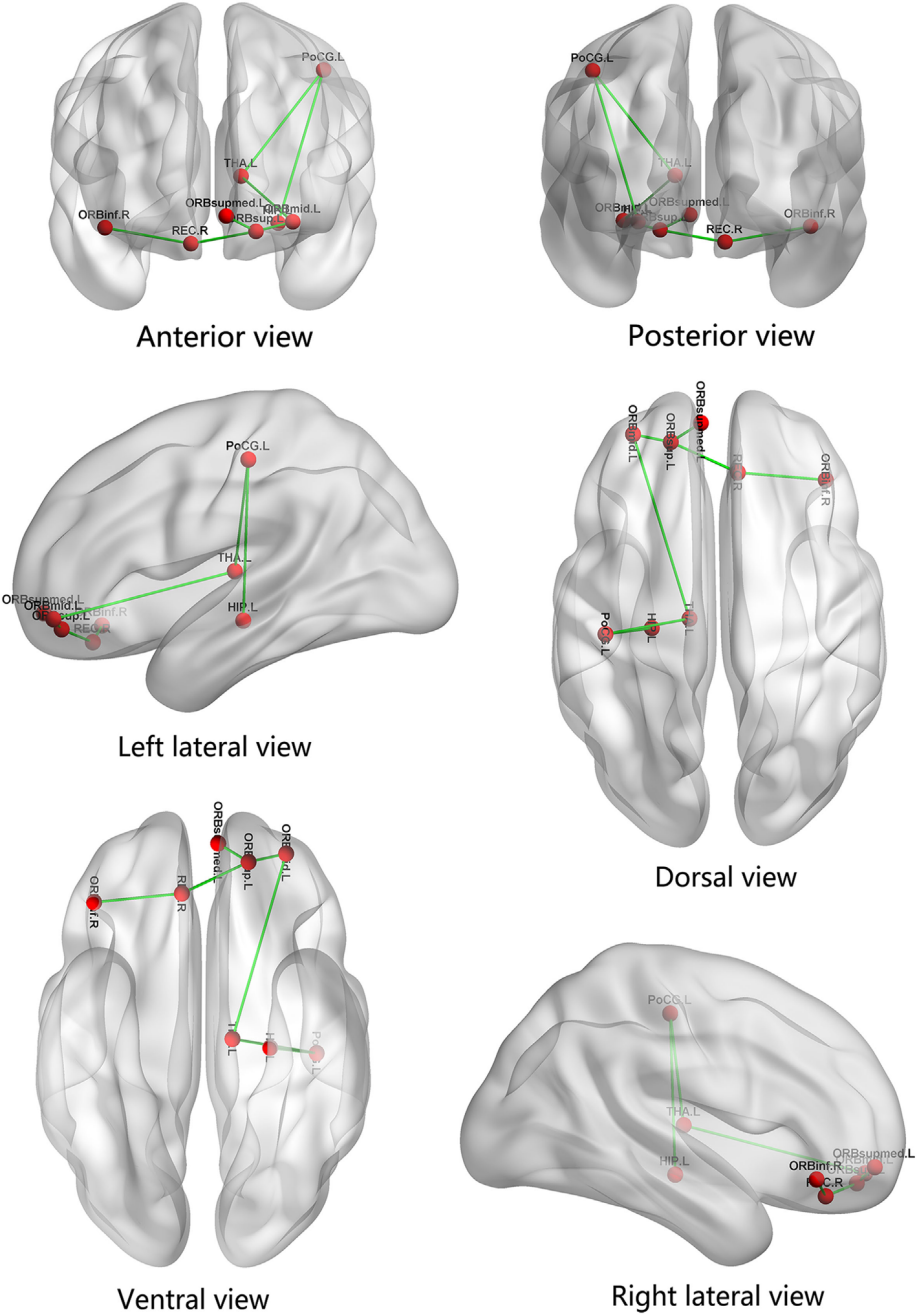
The significantly decreased subnetwork components are represented. The subnetwork consists of eight nodes and seven edges, mainly involving the frontal–subcortical and limbic regions. ORBmid.L, the left orbital part of middle frontal gyrus; ORBsup.L, the left orbital part of superior frontal gyrus; ORBsupmed.L, the left medial orbital part of superior frontal gyrus; THA.L, left thalamus; HIP.L, left hippocampus; PoCG.L, left postcentral gyrus; REC.R, right rectus gyrus; ORBinf.R, the right orbital part of inferior frontal gyrus.

Compared with the HCs, the MDD patient group presented with a significantly decreased subnetwork, which consisted of seven edges and eight nodes, in the NBS analysis results (*p* < 0.05, NBS corrected, see Table S3 in Supplementary Material). The subnetwork mainly encompassed bilateral orbitofrontal gyrus (the left orbital part of middle frontal gyrus, ORBmid.L; the left orbital part and medial orbital part of superior frontal gyrus, ORBsup.L and ORBsupmed.L; right rectus gyrus, REC.R; the right orbital part of inferior frontal gyrus, ORBinf.R), left thalamus (THA.L), hippocampus (HIP.L), and postcentral gyrus (PoCG.L). There was no increased subnetwork component in MDD patients compared with HCs. The visualization of the connectivity alterations is shown in Figure 2.

### Correlations between Network Measures and Clinical Variables

No global or regional topological metrics was related to either disease severity or duration in the group of MDD patients. And no significant correlation between structural connections and illness duration were found in MDD patients. But the structural connections between left thalamus and postcentral gyrus (*r* = −0.479, *p* = 0.033 < 0.05) (see Figure S3A in Supplementary Material), as well as ORBmid.L and ORBsup.L (*r* = −0.504, *p* = 0.024 < 0.05) (see Figure S3B in Supplementary Material), demonstrated significantly negative correlations with HDRS scores in MDD patients. However, we noticed there were a few extreme values in the sample. To exclude the bias effect of these extreme values, we recalculated the correlation after deleting them. Then we found the connections between left thalamus and postcentral gyrus (*r* = −0.408, *p* = 0.085 > 0.05) (see Figure S4A in Supplementary Material), as well as ORBmid.L and ORBsup.L (*r* = −0.317, *p* = 0.2 > 0.05) (see Figure S4B in Supplementary Material), had no significant correlation with HDRS scores in MDD patients.

## Discussion

In the present study, the structural network alternations of WM were investigated in a homogenous group of first episode, short duration and untreated MDD patients as well as matched HCs using graph theoretical and NBS analyses. First, we observed MDD patients had a significant decrease in the small-worldness (σ). Second, NBS analysis results demonstrated that, compared with the HCs, the MDD patients presented a significantly decreased subnetwork, which consisted of bilateral orbitofrontal cortex (OFC), left thalamus, hippocampus, and postcentral gyrus.

The first important finding of our study is that the small-worldness (σ) reduced in first episode, short duration, and untreated MDD patients compared to the controls. In the human brain network, small-worldness (σ) is mainly determined by the ratio of γ and λ. A network would be considered as a small-world network, if it is greater than 1.0 (48, 49), with a higher value indicating a more optimized balance between local specialization and global integration (50). In our study, both MDD patients and HCs showed small-world topological character, and the significant decrease of small-worldness (σ) might imply a less optimized balance in MDD group. Meanwhile, the reduction in σ was predominantly due to the reduction of γ and/or the increase of λ. However, in the present study, we did not find the significant reduction of γ and/or increase of λ after FDR corrected. Actually, it might be related to the statistical threshold (*p* < 0.05, FDR corrected) we employed. And there was still a slight reduction in γ values between the two groups, and the *p* value was much closed to 0.05 after the FDR correction (*p* = 0.072). So the slight alternation in γ might be contributed to interpret the reduction of small-worldness (σ). Besides, γ reflects the degree of functional segregation. So the reduction of small-worldness (σ) may be mainly related to the disruption of functional segregation in MDD patients. Notably, there were still some inconsistent results found between our study and several previous studies. Most of functional and structural network studies found no significant difference in the global network measures (28, 29, 51–53). Besides, decreased *L*_p_, γ, and λ, as well as increased *E*_glob_ and *E*_local_ were found in an early adult-onset, first-episode, treatment-naive depression patients group (30). The other structural network studies found a reduced *E*_glob_ and an increased *L*_p_ in both geriatric depression group (31) and remitted geriatric depression group (54), and the *C*_p_ was also found reduced in geriatric depression group. The reasons for these differences might be attributable to heterogeneity of MDD patients, such as differences in medication status, age of onset, the number of episodes and duration (27). Because the patients are all with relatively short duration (<1 year), our results possibly reflect the very early abnormality of this stage. These inconsistent results together suggest the necessity of future studies in bigger or homogeneous samples.

The second major finding in the present study was a significantly decreased subnetwork existed in MDD patients, including bilateral OFC, left thalamus, postcentral gyrus, and hippocampus. Although our finding was consistent with most previous structural network studies showing the decreased subnetworks in MDD patients, several previous studies still reported increased connections in first-episode, medication-naive MDD patients (30, 55). The inconsistent findings may be due to the sample heterogeneity and/or using of different network node and edge definitions, illness durations. Nevertheless, this finding further complemented our previous morphological study in first-episode and untreated MDD patients, which revealed the relationship between the left thalamic shape changes and the ipsilateral hippocampus, amygdala, and OFC (56). Our results together strongly support the abnormal connectivity roughly involved the LCSPT network (15) or LCSTC circuits (17) in early stage depressive patients. According to previous studies, both of the two neural circuits play an important role in regulating mood and emotional affect (17, 57, 58). Dysfunction in LCSPT or LCSTC circuits has been implicated as playing a key role in MDD. Meanwhile, although inconsistent in the globus pallidus, the two circuits were both involved in limbic systems, striatum and thalamus, and frontal lobe. In the present study, the decreased subnetwork components were in accordance with the areas shared by the two circuits. But the range of structural network alternations in our study is smaller than these previous studies. The cause for this result may be related to the early stage of the disease.

Furthermore, the nodal efficiency reduction of left thalamus and OFC were found in the MDD group using the graph theoretical analysis in the present study. Most of previous structural network studies revealed that MDD patients had significant abnormalities in the thalamus and/or OFC (28–30, 59, 60). These findings suggested that the thalamus and OFC might be the key nodes in subnetwork components. According to previous studies, the orbitofontal cortex (OFC) is comprised of the orbital part of the superior, middle, and inferior frontal gyrus, the medial orbital part of the superior frontal gyrus, rectus gyrus, and olfactory cortex (61) and may play a crucial role in the emotion-processing and cognitive functions, such as social cognition and decision making which are notorious dysfunction in major depression (62–65). For the changes of the OFC, although robust pathological evidences are currently lacking, several studies had provided some insight into possible links between the changes of the OFC and depression. For example, a postmortem study found MDD patients had decrease in cortical thickness, neuronal size and density in the OFC (66). Some previous structural studies had revealed significant OFC volume decrease in patients with depression (67, 68). Meanwhile, several resting-state fMRI and PET studies also reported that MDD patients showed decreased ReHo and regional cerebral blood flow in the OFC (69–72). Besides, Chen et al. revealed that the connectome alterations in the OFC among MDD patients may result from abnormalities in brain regional volume, WM tract integrity, and functional connectivity between brain structures (60). Consequently, the structural network alternations of the OFC might be related to the cognitive and emotion dysregulation in MDD patients.

Except for the OFC, thalamus is also considered as an integral part of the emotional salience network, emotion modulation network, and cognitive/executive network (73), as well as a complex sensory information node constituted by many nuclei (12). A recent meta-analysis of fMRI indicated that MDD patients exhibited abnormal activation in thalamus during the affective processing task (74). And several recent volumetric and advanced VBM studies observed a volume reduction of the thalamus in MDD patient, which may account for deficits in top-down regulation of negative affect (12, 67). Coincidentally, we also found MDD patients had the shape and volumetric changes in the thalamus at the early stage, which is negatively correlated with the severity of disease in our published study (56). Therefore, the abnormal variations of thalamus in our study may be implicated in pathological process of MDD. These structural and functional abnormalities of thalamus might be considered as potential markers of MDD. In addition, in the present study, as the WM structural network was constructed by weighted number and FA value of WM fibers, these alternations of thalamus might also attribute to the changes of WM FN or FA values in these regions. Coincidentally, Korgaonkar et al. observed that the connections between thalamus and other brain regions reduced in MDD patients when compared with nonpsychiatric subjects (28). They also found that MDD patients had a reduction in average FA value of the thalamic projection fibers in a TBSS-based DTI analysis (75). The result of these two studies from same team above were in accordance with our findings.

On the other hand, thalamus had been considered to play an important role in emotional and executive functions (67, 73), mainly due to its connections with OFC, anterior cingulate cortex (76), and the amygdala (77, 78). But a lot more researches showed that the thalamus and OFC both were parts of the reward circuit (79). Therefore, the structural connection alternations between the thalamus and OFC might help interpret the emotional and executive dysfunctions of depression.

Notably, the structural connection changes among the left thalamus, postcentral gyrus and hippocampus were also found in MDD group. According to the previous research, postcentral gyrus was thought to be the part of sensorimotor network (80) which was an important biomarker reflects the clinical psychomotor symptoms of MDD, such as psychomotor agitation or retardation (81, 82). So, the structural connection alternations between the thalamus and postcentral gyrus might related to clinical psychomotor symptoms.

Hippocampal volume reduction in patients with MDD is one of the most replicated findings confirmed by several meta-analysis of MRI morphological studies (83–85). Hippocampus plays a distinct role in the pathophysiology of MDD, mainly due to its sensitivity to stress (86). And stress, possibly acting *via* glucocorticoids, may negatively affect hippocampal volumes (87, 88). Despite the lack of robust evidence, the structural connection alternations of hippocampus may also result from the stress in depression. In addition, the hippocampus was also involved in cognitive functions and the regulations of emotion processes (89). For the cognitive deficit in MDD, it is suggested that hippocampus might play a critical role in memory deficit symptom of depressive patients (90). Persistent hypersecretion of glucocorticoids may contributes to hippocampal volume changes and cognitive dysfunctions in MDD patients, through neurotoxic effects on the hippocampus (91). What noteworthy is that the functional network disruptions in hippocampus was found to increase memory sensitivity to negative stimuli in MDD patient (92, 93). Therefore, the structural connectivity alternations of hippocampus may be related to the abnormal hypersecretion of glucocorticoids which lead to the cognitive and emotional dysfunctions in MDD patients.

Additionally, we only found the trends of negative structural connections between some regions with symptom severity in the MDD patients. These correlations disappeared after excluding a few extreme values in the sample. These results suggested that the contribution of alternations of structural connections on depression severity might further confirmation in the big sample in the future.

Several limitations of our study should be addressed. First, due to the relatively small sample size design, the results cannot be generalized to the general population. But through power analysis, the sample size in the present study met the minimum sample size of statistical requirements, so the statistical results were still reliable. The second limitation involves DTI technique in resolving crossing fibers and sharp angulations of tracts (94). This can lead to false-positive connections. Thus, high angular resolution diffusion imaging diffusion models should be favored, which are considered capable of resolving complex fiber crossings (95). Additionally, there are very few MDD patients without anxiety symptom, which is also the limitation in the present study. So next, the characteristics of MDD patients with or without comorbid anxiety disorders should be analyzed in a large sample. Finally, although we discussed the possible relationships among structural network changes, clinical psychomotor symptoms and cognition, the evaluation of clinical psychomotor symptoms and cognitive function was not carried out, and consequently, the exact relationships among structural network changes, clinical psychomotor symptoms and cognitive functions remain speculative and were not addressed here. Future studies with a larger sample size of first episode, short duration, untreated MDD are necessary and the relationship between clinical factors and neuroimaging results need to be clarified in the further researches.

## Conclusion

In total, our results suggested the abnormal structural network of the OFC and thalamus, involving the imbalance with the limbic system, as a key pathology in early stage drug-naive depression patients. Excluded the effect of chronic duration, medication, and multiple episode, these results might possibly reflect the trait characters of the disease at the very early stage.

## Ethics Statement

This study was carried out in accordance with the recommendations of ethic committee of kunming medical university with written informed consent from all subjects. All subjects gave written informed consent in accordance with the Declaration of Helsinki. The protocol was approved by the ethic committee of Kunming medical university.

## Author Contributions

DH designed experiments. YL, ZS, and YC carried out experiments and analyzed experimental results. YL wrote the manuscript. HY assisted with statistical analysis. YX, LW, BH, ZZ, XS, WZ, and XX assisted with carrying out experiments.

## Conflict of Interest Statement

The authors declare that the research was conducted in the absence of any commercial or financial relationships that could be construed as a potential conflict of interest.

## Footnotes

^1^http://www.nitrc.org/projects/panda/.

^2^www.nitrc.org/projects/gretna.

^3^www.nitrc.org/projects/bnv.
