# Supplementary material for: Alternations of White Matter Structural Networks in First Episode Untreated Major Depressive Disorder with Short Duration
Source: Front Psychiatry. 2017 Oct 25;8:205. doi: 10.3389/fpsyt.2017.00205 (PMC5661170; doi:10.3389/fpsyt.2017.00205)
Supplement: Supplementary file 1 [file Table_1.DOCX]

Supplementary Material

**Alternations of white matter structural networks in first episode, untreated major depressive disorder with short-duration**

Yi Lu *, Zonglin Shen*, Yuqi Cheng*, Hui Yang, Bo He, Yue Xie, Liang Wen, Zhenguang Zhang, Xuejin Sun, Wei Zhao, Xiufeng Xu

* Corresponding Author: Dan Han, kmhandan@sina.com

# Supplementary Tables

Supplementary Table 1.  90 cortical and subcortical regions in Automated Anatomical Labeling (AAL) template

| No | Regions | Abbr. | No | Regions | Abbr. |
| --- | --- | --- | --- | --- | --- |
| 1 | Left Precentral gyrus | PreCG.L | 2 | Right Precentral gyrus | PreCG.R |
| 3 | Left Superior frontal gyrus, dorsolateral | SFGdor.L | 4 | Right Superior frontal gyrus, dorsolateral | SFGdor.R |
| 5 | Left Superior frontal gyrus, orbital part | ORBsup.L | 6 | Right Superior frontal gyrus, orbital part | ORBsup.R |
| 7 | Left Middle frontal gyrus | MFG.L | 8 | Right Middle frontal gyrus | MFG.R |
| 9 | Left Middle frontal gyrus, orbital part | ORBmid.L | 10 | Right Middle frontal gyrus, orbital part | ORBmid.R |
| 11 | Left Inferior frontal gyrus, opercular part | IFGoperc.L | 12 | Right Inferior frontal gyrus, opercular part | IFGoperc.R |
| 13 | Left Inferior frontal gyrus, triangular part | IFGtriang.L | 14 | Right Inferior frontal gyrus, triangular part | IFGtriang.R |
| 15 | Left Inferior frontal gyrus, orbital part | ORBinf.L | 16 | Right Inferior frontal gyrus, orbital part | ORBinf.R |
| 17 | Left Rolandic operculum | ROL.L | 18 | Right Rolandic operculum | ROL.R |
| 19 | Left Supplementary motor area | SMA.L | 20 | Right Supplementary motor area | SMA.R |
| 21 | Left Olfactory cortex | OLF.L | 22 | Right Olfactory cortex | OLF.R |
| 23 | Left Superior frontal gyrus, medial | SFGmed.L | 24 | Right Superior frontal gyrus, medial | SFGmed.R |
| 25 | Left Superior frontal gyrus, medial orbital | ORBsupmed.L | 26 | Right Superior frontal gyrus, medial orbital | ORBsupmed.R |
| 27 | Left Rectus gyrus | REC.L | 28 | Right Rectus gyrus | REC.R |
| 29 | Left Insula | INS.L | 30 | Right Insula | INS.R |
| 31 | Left Anterior cingulate and paracingulate gyrus | ACG.L | 32 | Right Anterior cingulate and paracingulate gyrus | ACG.R |
| 33 | Left Median cingulate and paracingulate gyrus | DCG.L | 34 | Right Median cingulate and paracingulate gyrus | DCG.R |
| 35 | Left Posterior cingulate gyrus | PCG.L | 36 | Right Posterior cingulate gyrus | PCG.R |
| 37 | Left Hippocampus | HIP.L | 38 | Right Hippocampus | HIP.R |
| 39 | Left Parahippocampal gyrus | PHG.L | 40 | Right Parahippocampal gyrus | PHG.R |
| 41 | Left Amygdala | AMYG.L | 42 | Right Amygdala | AMYG.R |
| 43 | Left Calcarine fissure and surrounding cortex | CAL.L | 44 | Right Calcarine fissure and surrounding cortex | CAL.R |
| 45 | Left Cuneus | CUN.L | 46 | Right Cuneus | CUN.R |
| 47 | Left Lingual gyrus | LING.L | 48 | Right Lingual gyrus | LING.R |
| 49 | Left Superior occipital gyrus | SOG.L | 50 | Right Superior occipital gyrus | SOG.R |
| 51 | Left Middle occipital gyrus | MOG.L | 52 | Right Middle occipital gyrus | MOG.R |
| 53 | Left Inferior occipital gyrus | IOG.L | 54 | Right Inferior occipital gyrus | IOG.R |
| 55 | Left Fusiform gyrus | FFG.L | 56 | Right Fusiform gyrus | FFG.R |
| 57 | Left Postcentral gyrus | PoCG.L | 58 | Right Postcentral gyrus | PoCG.R |
| 59 | Left Superior parietal gyrus | SPG.L | 60 | Right Superior parietal gyrus | SPG.R |
| 61 | Left Inferior parietal gyrus | IPL.L | 62 | Right Inferior parietal gyrus | IPL.R |
| 63 | Left Supramarginal gyrus | SMG.L | 64 | Right Supramarginal gyrus | SMG.R |
| 65 | Left Angular gyrus | ANG.L | 66 | Right Angular gyrus | ANG.R |
| 67 | Left Precuneus | PCUN.L | 68 | Right Precuneus | PCUN.R |
| 69 | Left Paracentral lobule | PCL.L | 70 | Right Paracentral lobule | PCL.R |
| 71 | Left Caudate nucleus | CAU.L | 72 | Right Caudate nucleus | CAU.R |
| 73 | Left Lenticular nucleus, putamen | PUT.L | 74 | Right Lenticular nucleus, putamen | PUT.R |
| 75 | Left Lenticular nucleus, pallidum | PAL.L | 76 | Right Lenticular nucleus, pallidum | PAL.R |
| 77 | Left Thalamus | THA.L | 78 | Right Thalamus | THA.R |
| 79 | Left Heschl gyrus | HES.L | 80 | Right Heschl gyrus | HES.R |
| 81 | Left Superior temporal gyrus | STG.L | 82 | Right Superior temporal gyrus | STG.R |
| 83 | Left Temporal pole: superior temporal gyrus | TPOsup.L | 84 | Right Temporal pole: superior temporal gyrus | TPOsup.R |
| 85 | Left Middle temporal gyrus | MTG.L | 86 | Right Middle temporal gyrus | MTG.R |
| 87 | Left Temporal pole: middle temporal gyrus | TPOmid.L | 88 | Right Temporal pole: middle temporal gyrus | TPOmid.R |
| 89 | Left Inferior temporal gyrus | ITG.L | 90 | Right Inferior temporal gyrus | ITG.R |
