# Supplementary material for: Alternations of White Matter Structural Networks in First Episode Untreated Major Depressive Disorder with Short Duration
Source: Front Psychiatry. 2017 Oct 25;8:205. doi: 10.3389/fpsyt.2017.00205 (PMC5661170; doi:10.3389/fpsyt.2017.00205)
Supplement: Supplementary file 2 [file Table_2.DOCX]

Supplementary Material

**Alternations of white matter structural networks in first episode, untreated major depressive disorder with short-duration**

First-author:Yi Lu *, Zonglin Shen*, Yuqi Cheng*, Hui Yang, Bo He, Yue Xie, Liang Wen, Zhenguang Zhang, Xuejin Sun, Wei Zhao, Xiufeng Xu

* Corresponding Author: Dan Han, kmhandan@sina.com

# Supplementary Tables

Supplementary Table 2. Alterations of global network measures

|  | Global network measures | FDR corrected *p* value |
| --- | --- | --- |
| 1 | C_p_ (clustering coefficient) | 0.46 |
| 2 | L_p_ (characteristic path length) | 0.53 |
| 3 | γ (normalized clustering coefficient) | 0.071 |
| 4 | λ (normalized characteristic path length) | 0.68 |
| 5 | E_glob_ (global efficiency) | 0.70 |
| 6 | E_loc_ (local efficiency) | 0.71 |
