# Supplementary material for: Alternations of White Matter Structural Networks in First Episode Untreated Major Depressive Disorder with Short Duration
Source: Front Psychiatry. 2017 Oct 25;8:205. doi: 10.3389/fpsyt.2017.00205 (PMC5661170; doi:10.3389/fpsyt.2017.00205)
Supplement: Supplementary file 3 [file Table_3.DOCX]

Supplementary Material

**Alternations of white matter structural networks in first episode, untreated major depressive disorder with short-duration**

First-author:Yi Lu *, Zonglin Shen*, Yuqi Cheng*, Hui Yang, Bo He, Yue Xie, Liang Wen, Zhenguang Zhang, Xuejin Sun, Wei Zhao, Xiufeng Xu

* Corresponding Author: Dan Han, kmhandan@sina.com

# Supplementary Tables

Supplementary Table 3. NBS results

|  | Connection | Test statistics |
| --- | --- | --- |
| 1 | Frontal_Sup_Orb_L to Frontal_Mid_Orb_L | 2.14 |
| 2 | Frontal_Sup_Orb_L to Frontal_Med_Orb_L | 2.26 |
| 3 | Frontal_Sup_Orb_L to Rectus_R | 2.64 |
| 4 | Frontal_Inf_Orb_R to Rectus_R | 2.27 |
| 5 | Hippocampus_L to Postcentral_L | 2.21 |
| 6 | Frontal_Mid_Orb_L to Thalamus_L | 2.27 |
| 7 | Postcentral_L to Thalamus_L | 2.39 |
