# Supplementary figures and images for: Alternations of White Matter Structural Networks in First Episode Untreated Major Depressive Disorder with Short Duration
Source: Front Psychiatry. 2017 Oct 25;8:205. doi: 10.3389/fpsyt.2017.00205 (PMC5661170; doi:10.3389/fpsyt.2017.00205)

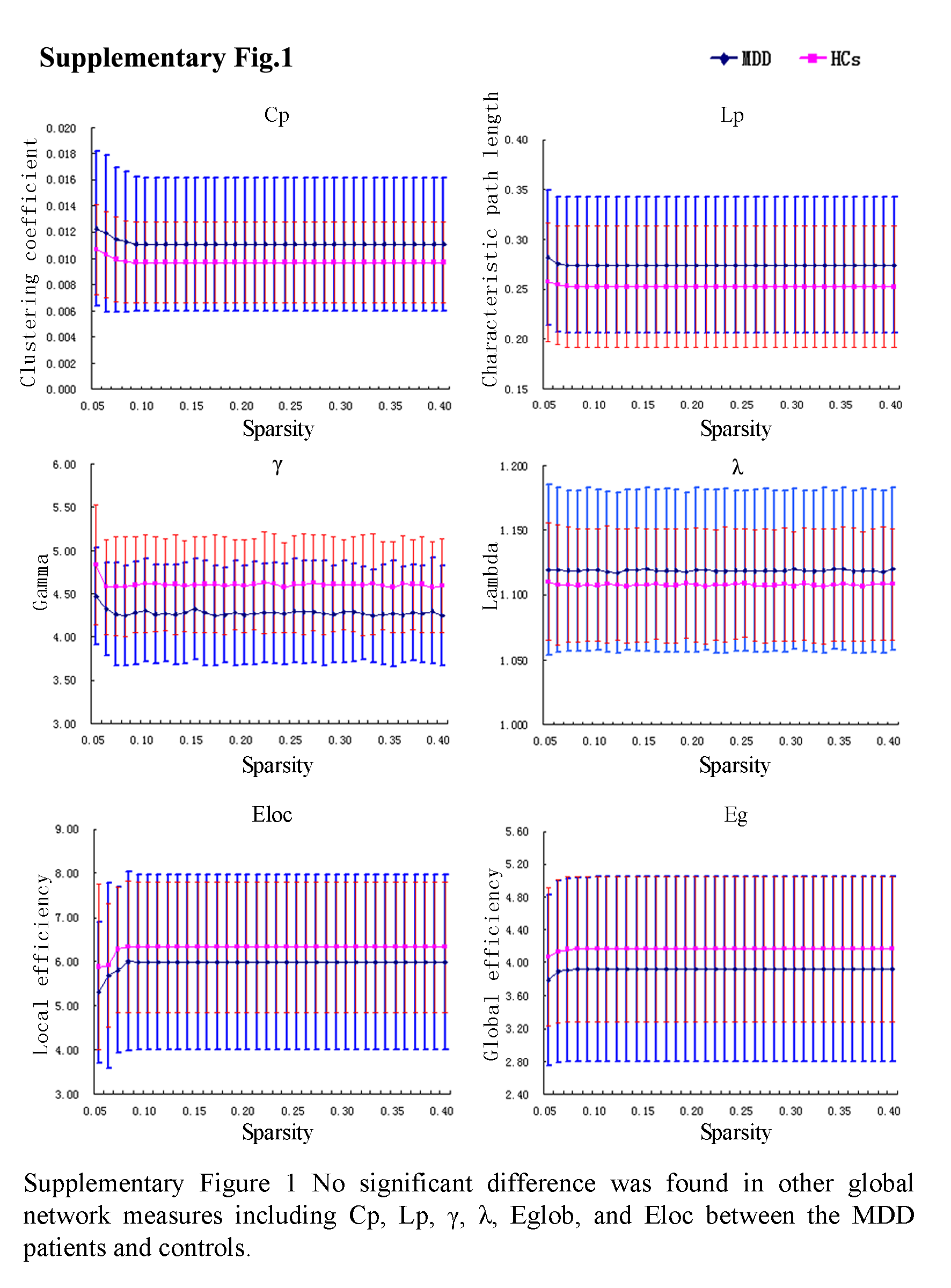

Supplement: Supplementary file 4 [file Image_1.TIF]

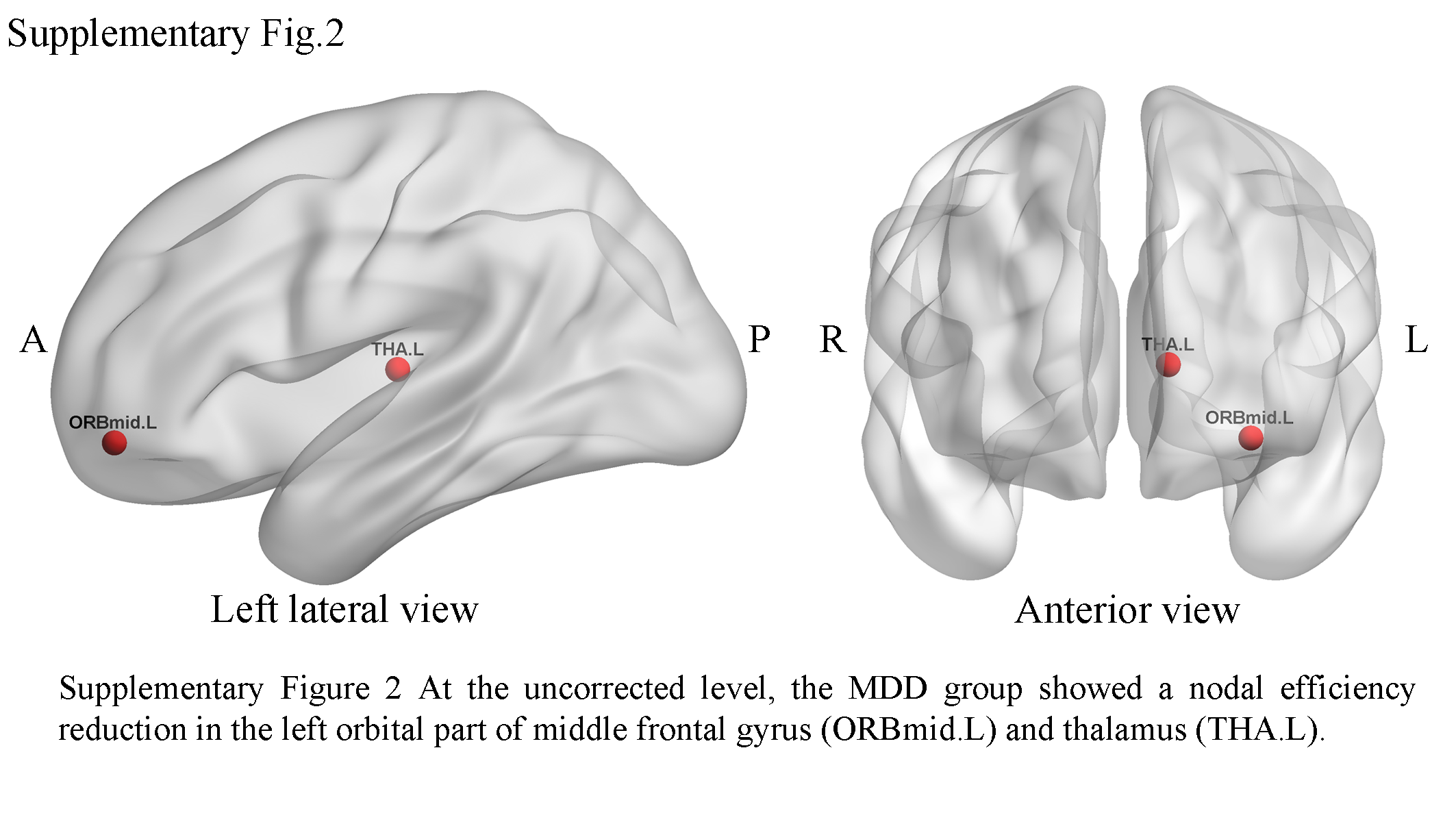

Supplement: Supplementary file 5 [file Image_2.TIF]

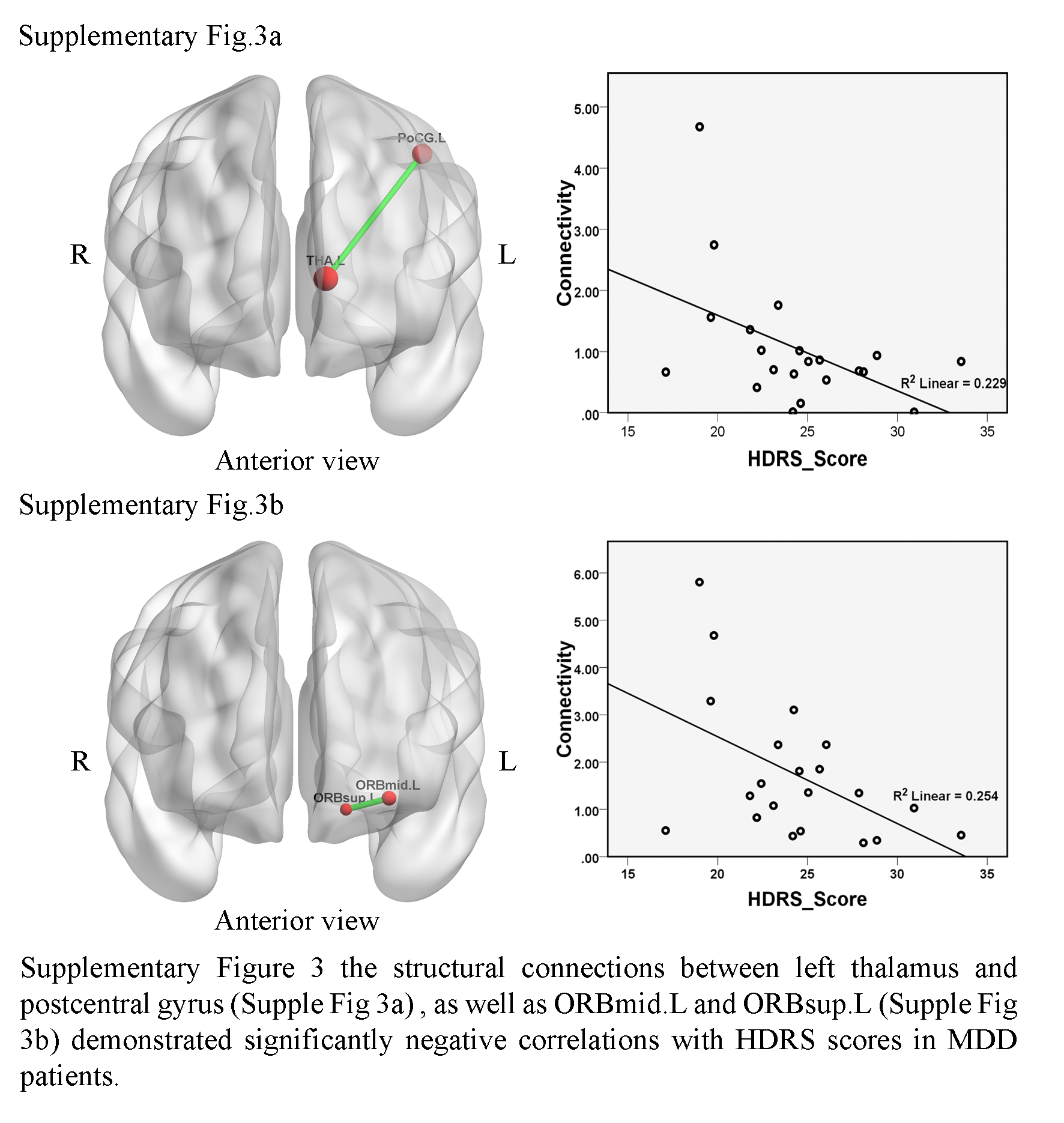

Supplement: Supplementary file 6 [file Image_3.TIF]

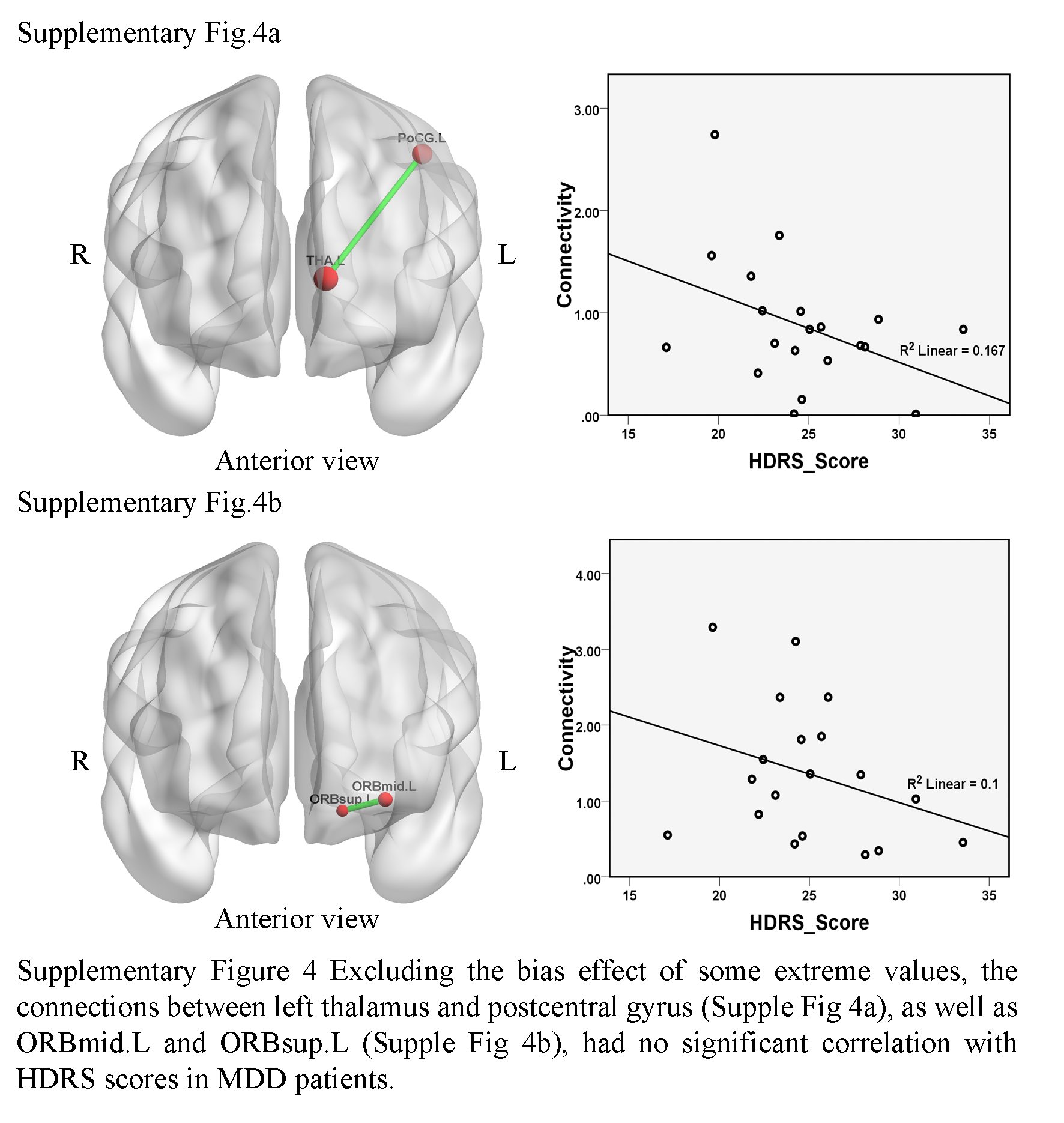

Supplement: Supplementary file 7 [file Image_4.TIF]
